# Mindfulness-based cognitive therapy vs. a health enhancement program for the treatment of late-life depression: Study protocol for a multi-site randomized controlled trial

**DOI:** 10.3389/fnagi.2022.976636

**Published:** 2022-09-01

**Authors:** Magnus Bein, Myriam Lesage, Elena Dikaios, Mallar Chakravarty, Zindel Segal, Isabelle Royal, Mark Speechley, Alessandra Schiavetto, Daniel Blumberger, Matthew D. Sacchet, Joseph Therriault, Johanna Gruber, Valerie Tourjman, Stephane Richard-Devantoy, Vasavan Nair, Marie-Andrée Bruneau, Soham Rej, Michael Lifshitz, Harmehr Sekhon

**Affiliations:** ^1^Department of Psychiatry, GeriPARTy Research Group, Jewish General Hospital, Montréal, QC, Canada; ^2^Departments of Biological and Biomedical Engineering and Psychiatry, Centre d'imagerie cérébrale, Douglas Mental Health Institute, Verdun, QC, Canada; ^3^University of Toronto–Scarborough, Toronto, ON, Canada; ^4^Neuropsychology Service, Department of Psychiatry, Jewish General Hospital, Montréal, QC, Canada; ^5^Department of Epidemiology and Biostatistics, University of Western Ontario, London, ON, Canada; ^6^Department of Psychiatry, Jewish General Hospital, McGill University, Montréal, QC, Canada; ^7^Department of Psychiatry, Centre for Addiction and Mental Health, University of Toronto, Toronto, ON, Canada; ^8^Meditation Research Program, Department of Psychiatry, Harvard Medical School, Massachusetts General Hospital, Boston, MA, United States; ^9^Department of Neurology and Neurosurgery, Translational Neuroimaging Laboratory, McGill Research Centre for Studies in Aging, Douglas Mental Health Institute, Le Centre intégré universitaire de santé et de services sociaux (CIUSSS) de l'Ouest de l'Île de Montréal, Montréal, QC, Canada; ^10^Department of Psychiatry, Institut Universitaire en Santé Mentale de Montréal, Montréal, QC, Canada; ^11^Department of Psychiatry, Douglas Mental Health Institute, Verdun, QC, Canada; ^12^Département de psychiatrie et d'addictologie, Research Centre, Institut Universitaire de Gériatrie de Montréal, Montréal, QC, Canada; ^13^Division of Social and Transcultural Psychiatry, Department of Psychiatry, McGill University, Montréal, QC, Canada; ^14^Division of Geriatric Psychiatry, Harvard Medical School, McLean Hospital, Cambridge, MA, United States

**Keywords:** late-life depression, mindfulness, cognitive function, memory, behavioral activation, executive function, processing speed

## Abstract

**Background:**

Late-life depression (LLD) affects up to 18% of older adults and has been linked to elevated dementia risk. Mindfulness-based cognitive therapy (MBCT) holds promise for treating symptoms of depression and ameliorating cognitive deficits in older adults. While preliminary findings are promising, a definitive RCT investigating its effects on late life depression and cognition have not yet been conducted. We present a protocol describing a multi-site blinded randomized controlled trial, comparing the effects of MBCT and of an active control, a Health Enhancement Program (HEP), on depressive symptoms, executive functioning, and brain biomarkers of LLD, among several other exploratory outcomes.

**Methods:**

Two-hundred and thirteen (*n* = 213) patients with LLD will be recruited at various centers in Montreal, QC, Canada. Participants will undergo stratified randomization to either MBCT or HEP intervention groups. We will assess changes in (1) depression severity using the Hamilton Depression Rating Scale (HAM-D17), (2) processing speed and executive functioning, (3) brain biomarkers of LLD (hippocampal volume, default network resting-state functional connectivity and executive network resting-state functional connectivity), and (4) other exploratory physiological and mood-based measures, at baseline (0 weeks), post intervention (8 weeks), and 26 weeks after baseline.

**Discussion:**

The proposed study will assess the clinical potential of MBCT to improve symptoms of depression, as well as examine its impact on cognitive impairments and neurobiological markers, and thus inform its use as a promising adjunct in the treatment of LLD.

**Clinical trial registration:**

www.ClinicalTrials.gov, identifier: NCT05366088.

## Background

With a global prevalence between 9 and 18% (Luijendijk et al., 2008), late life depression (LLD) affects over 300,000 new Canadian seniors with $5 billion in direct health-care costs (Vasiliadis et al., 2013). LLD contributes to increased health-care utilization (Vasiliadis et al., 2013; Sivertsen et al., 2015), decreased quality of life (Sivertsen et al., 2015) and caregiver burden (Vasiliadis et al., 2013), and is associated with a 2-fold increased risk of physical comorbidity and mortality (Grover et al., 2018; Wei et al., 2019). An estimated 60% of patients with LLD will be resistant to treatment-as-usual (TAU), antidepressants and psychotherapy (Knochel et al., 2015). Furthermore, Antidepressants are discontinued in 30% of older adults due to tolerability issues (Tham et al., 2016; Lindblad et al., 2019).

Cognitive functioning deficits are found in 20–50% of people with LLD (Koenig et al., 2014a), and cognitive changes remain resistant to treatments in ≥30% of these individuals (Lee et al., 2007; Bhalla et al., 2009). In particular, processing speed and executive function deficits have been associated with poor treatment response (Story et al., 2008; Jungwirth et al., 2011; Pimontel et al., 2012; Cristancho et al., 2018; Rutherford et al., 2021). The most consistently replicated brain biomarkers of LLD are hippocampal atrophy (Du et al., 2014; Taylor et al., 2014) and brain network dysregulation (Tadayonnejad and Ajilore, 2014). This pattern of hippocampal atrophy and altered functional connectivity is linked to elevated dementia risk in LLD (Rashidi-Ranjbar et al., 2020; Schwab et al., 2020).

Mindfulness-based cognitive therapy (MBCT) targets specific brain circuits implicated in memory and attention regulation and holds promise for ameliorating cognitive deficits. Mindfulness interventions have been shown to increase cognitive functioning (Berk et al., 2017) functional connectivity of the default network (Sevinc et al., 2021) and gray matter concentration in the hippocampus in as little as 4-weeks in older adults (Greenberg et al., 2019). Pilot studies have also demonstrated reductions in depressive symptoms following MBCT treatment with medium to large effect sizes, comparable to first-line antidepressants and individual psychotherapy (Barnhofer et al., 2009; MacKenzie and Kocovski, 2016; Torres-Platas et al., 2019; Musa et al., 2020).

While preliminary findings are promising, a definitive RCT investigating its effects on late life depression and cognition has not yet been conducted. This study will compare the effects of MBCT vs. an active control group, Health Enhancement Program (HEP) on symptoms of LLD as well as memory and cognition and brain structure and function.

## Methods

### Study design

A multi-site phase III RCT with 213 LLD patients, randomized 1:1 to MBCT or HEP. This will be an 8-week two-arm, assessor-blind RCT of MBCT vs. HEP in LLD, at various centers in Montreal. Participants will attend three assessment sessions (baseline/0 weeks, 8 weeks, and 26 weeks) in a research-hospital setting. Participants will attend either MBCT or HEP during group sessions for 2 h/week, for 8 weeks. We will assess change in:

1. Depression scores (HAM-D17) in the MBCT arm compared to HEP at 8 weeks. We will also assess whether participants in the MBCT arm will continue to have reduced depression scores at the 26-week follow-up (Primary outcome).
2. Executive functioning and processing speed in the MBCT arm compared to HEP at 8 weeks. We will also assess whether participants in the MBCT arm will continue to have improved cognitive scores at the 26-week follow-up (Secondary outcome A).
3. Hippocampal volume, default network resting-state functional connectivity and executive network resting-state functional connectivity at 8 weeks. We will also assess whether participants in the MBCT arm will maintain greater increases in hippocampal volume, increases in default network resting-state functional connectivity, and decreases in executive network resting-state functional connectivity at the 26-week follow-up (Secondary outcome B).

### Interventions

**Figure 1:**
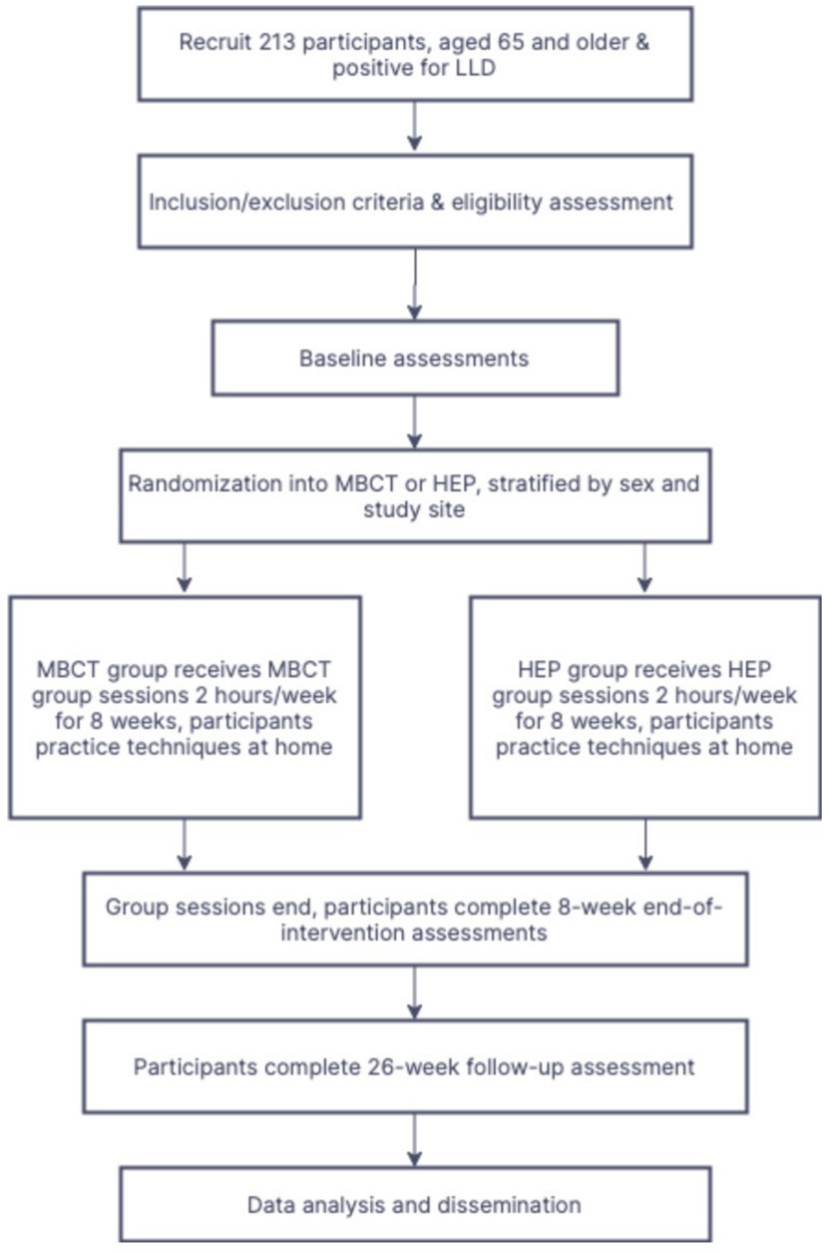
Participant Allocation Flowchart.

Participants will be randomized to Mindfulness-Based Cognitive Therapy (MBCT) or Health Enhancement Program (HEP) for 8-weeks, in addition to their usual antidepressant therapy and/or psychosocial care. MBCT will be delivered by licensed MBCT clinicians, while HEP will be delivered by trained HEP teachers which meet the *Qualifications and Recommended Guidelines for HEP Providers*, outlined in the HEP Guidelines (Sullivan et al., 2011). See Figure 1 for an overview of the allocation of participants and the study timeline.

#### Mindfulness-based cognitive therapy

MBCT is an 8-week therapy integrating formal mindfulness meditation (e.g., breath and body awareness) and informal mindfulness (e.g., eating, walking). Participants are taught to attend non-judgmentally to present moment experiences. MBCT includes cognitive therapy techniques to target the ruminative thought processes and identification with negative emotions seen in depression (Segal et al., 2002). MBCT teaches participants how to disengage from habitual (“automatic”) dysfunctional cognitive routines, in particular depression-related ruminative thought patterns that perpetuate depressive symptoms and increase vulnerability to relapse (Segal et al., 2018).

The program consists of group sessions 2 h/week, for 8 weeks, and will be adapted to older adults to promote comfort and safety, e.g., decreasing sitting meditation time (max. 20 min) and modifying postures. Brief discussions will reinforce principles of mindfulness: awareness, non-judgment and acceptance. Participants will be provided with meditation recordings to facilitate home practice (~30 min/day, 6 days/week). Interventionists will meet regularly, to harmonize their curriculum/geriatric modifications of MBCT. Fidelity of intervention will be assessed by asking facilitators which elements of MBCT they covered in each session. Additionally, an independent assessor will measure fidelity of intervention by administering the Mindfulness-Based Interventions-Teaching Assessment Criteria (Crane et al., 2013; Evans et al., 2021).

#### Health-enhancement program

HEP teaches health-enhancing techniques and was designed by University of Wisconsin and NIH as a manualized active control group program for mindfulness-based intervention trials (Sullivan et al., 2011; MacCoon et al., 2012). We have tailored HEP to be structurally equivalent to MBCT, with similar-sized groups, meeting weekly for 2 h for 8 weeks, and similar amount of home practice (~30 min/day, 6 days/week). HEP controls for several non-specific factors in mindfulness groups, including: group support/morale, behavioral activation, stigma reduction, facilitator attention, treatment duration, and time spent on home practice. Participants will learn about health promotion, healthy diet, music, and gentle exercise, but not about breathing techniques or meditation.

### Participants

We will recruit 213 participants, from various primary care centers and academic and community mental health clinics associated with our three sites: McGill, Université de Montréal, and University of Toronto.

Inclusion criteria: (1) aged 60–85 years of age with major depressive disorder as assessed by the Mini International Neuropsychiatric Inventory [MINI; (Hainline et al., 2018)] and verified by a psychiatrist; (2) HAM-D17 score ≥ 10 (Hamilton, 1960); (3) participants willing and able to attend ≥75% of MBCT/HEP sessions; (4) sufficient hearing to follow verbal instructions; (5) adequate understanding of English and/or French as groups will be run in English and French; and (6) ability to sit for 40 min without discomfort.

Exclusion criteria: (1) inability to provide informed consent; (2) evidence of dementia as defined by MoCA score >19 (Nasreddine et al., 2005; Dautzenberg et al., 2020); (3) lifetime diagnosis of bipolar I or II disorder, primary psychotic disorder (e.g., schizophrenia, schizoaffective disorder), and/or severe personality disorder interfering with ability to function in a group; (4) substance abuse/dependence within the past 6 months; (5) high suicide risk (e.g., active suicidal ideation and/or current/recent intent or plan); (6) non-correctable, clinically significant sensory impairment; (7) significant impairments in fine motor skills; (8) acutely unstable medical illnesses, including delirium, acute cerebrovascular/cardiovascular events within the last 6 months; having a terminal medical diagnosis with prognosis of <12 months; (9) currently practicing any mind-body intervention on a regular basis; (10) unwilling or unable to remain on the same psychotropic medications (includes dosage) for the first 8 weeks of the study; and (11) any of the following contraindications for a magnetic resonance study: pacemaker, aneurysm clip, heart/vascular clip, prosthetic valve, metal prosthesis, claustrophobia, metal fragments in body, and transdermal patches.

#### Randomization

Participants will undergo 1:1 simple randomization to the intervention (MBCT) or the active control (HEP), stratified by sex and study site to control for unequal numbers of participants and differential efficacy between sites. Raters and clinicians will be blinded to participant group allocation and participants will be blinded to study hypotheses. We will offer the MBCT and HEP in groups of 8–11 participants recruited on a rolling basis.

### Outcomes

A screening assessment will be conducted to determine a participant's eligibility for the study. Following completion of the informed consent process, baseline assessments will be completed. A medication record will be collected as per participant report along with reconciliation from pharmacy records. Outcomes will be measured at initial, week 8 (primary study endpoint), and at week 26 follow-up stages (maintenance effects). The following scales will be administered at the baseline assessments.

#### Primary outcome

##### Hamilton depression rating scale (HAM-D17) scores

The HAM-D17 is a well-validated assessor-rated scale used in LLD trials (Koenig et al., 2014b; Lavretsky et al., 2015). HAM-D17 includes 17 items, scored 0–4, with a total score of 0–52: Scores ≥24, 17–23, 8–16, and 0–7 indicate severe, moderate, mild, and no depression, respectively.

#### Secondary outcomes

##### Cognitive testing scores

Processing speed [Trail Making Test A (Reitan, 1956) and WAIS-IV Digit Symbol (Wechsler, 2012)] and executive functioning [Trail Making Test B (Reitan, 1956), WAIS- IV Digit Span (Wechsler, 2012), Emotional Stroop Task (Williams et al., 1996), and Verbal Fluency Test (Lezak et al., 2004)] domain Z-scores will be calculated using a neurocognitive battery, conducted by trained assessors, at baseline, 8-weeks and 26-week follow up; and global cognition [exploratory; Montreal Cognitive Assessment (MoCA)] will be calculated at baseline, 8- and 26- week follow-up.

##### Brain biomarkers

Functional and structural magnetic resonance imaging (MRI) will be collected from a subset of the overall sample (*n* = 40) at Montreal sites (McGill and UdeM) at baseline, 8-weeks, and 26-weeks in a 3T Siemens Prisma scanner. All imaging data will be acquired at the Douglas Institute site to avoid known issues of scan-site variability (Hainline et al., 2018). Based on our experience collecting fMRI data with geriatric patients, we have allotted 60 min scan-time for each assessment (including setup).

##### Hippocampal volume

T1 weighted MPRAGE will be acquired (TE/TR = 2.98 ms/2,300 ms, TI = 900 ms, α = 9°, FOV = 256 x 240 x 192 mm, GRAPPA of 2, 5 min). Volumetry of the hippocampus and its subfields will be identified using the MAGeT Brain algorithm (Chakravarty et al., 2013; Pipitone et al., 2014). MAGeT Brain improves segmentation over traditional model-based techniques by bootstrapping the segmentation and provides surface-based morphology measures. Cortical thickness will be derived using CIVET version 2.1; (Ad-Dab'bagh et al., 2006) T1-weighted images are pre-processed, tissue classified, white and gray matter surface extracted, and thickness measures are blurred using a 20-mm surface-based kernel.

##### Resting-state functional connectivity

Resting-state functional MRI data will be acquired using simultaneous multislice echo planar imaging sequence (Miller et al., 2015) with a scan time of ~5 mins, with the following parameters: TE/TR = 30 ms/1,000 ms, 300 frames, slice thickness = 2.5 mm, and 2.5 mm isotropic in-plane resolution, and slice acceleration factor (SMS) of 4. A matching B0 field map will be acquired with an approximate scan time of 1.5 min, with the following parameters: TE 1 = 4.92 ms, TE 2 = 7.38 ms, TR = 688 ms.

#### Exploratory outcomes

We will collect the following exploratory outcomes at baseline, 8-week, and 26-weeks: quality of Life [EQ-5D; (Herdman et al., 2011)], anxiety [GAD-7; (Spitzer et al., 2006)], mindfulness [FFMQ-SF; (Baer et al., 2006)], global cognition [MoCA; (Nasreddine et al., 2005)], general vascular burden [CIRS-G vascular subscale; (Linn et al., 1968)], and brain iron accumulation/microbleeds *via* quantitative susceptibility mapping. A detailed description of exploratory outcomes can be found in Table 1.

**Table 1:** Exploratory outcome measures for all participants, administered at 0 weeks (baseline), 8 weeks (end of intervention) and 26 weeks (follow-up).

**Exploratory outcomes**	**Description**
EuroQol-5D	The EQ-5D is a well-validated quality of life scale used in clinical depression trials. Research has shown that a patient's quality of life influences their level of help-seeking and treatment adherence. As such, it is important to examine patients' assessment of their own wellbeing and functioning.
Generalized anxiety disorder questionnaire (GAD-7)	The GAD-7 is a brief self-report measure of anxiety which is useful to measure in this population given the high comorbidity of depression and anxiety in late life, as well as the associated lower quality of life and poorer treatment response seen with anxiety.
Short-form of the five facet mindfulness questionnaire (FFMQ-SF)	The FFMQ-SF has been found to be a valid and reliable measure of dispositional mindfulness in depressed older adults in assisted care. We chose the short form to reduce participant burden.
The montreal cognitive assessment (MoCA)	The MoCA examines seven different cognitive domains, including visuospatial/executive functioning; naming; attention/concentration; language; abstraction; delayed recall; and orientation in time and place. The MoCA will be used to screen out patients with dementia who may be less likely to benefit from this study (MoCA score <19), and to measure global cognition at baseline, 8-week, and 26-week time points.
Cumulative illness rating scale-geriatric (CIRS-G) vascular subscale	The Cumulative Illness Rating Scale (CIRS-G) is a user-friendly but comprehensive review of common medical problems of the elderly by organ system. The vascular subscale focuses on common vascular issues (e.g., hypertension, atherosclerosis disease, history of aortic aneurysm, etc.). We chose to include the CIGS-R vascular subscale because vascular risk factors can account for mood disorder and functional impairment among geriatric patients.
Quantitative susceptibility mapping (QSM) assessment based on T2^*^-weighted imaging	T2^*^ and magnetic susceptibility mapping uses a multi-echo gradient echo sequence which is differentially sensitive to myelin and iron content, and consequently provides an important marker for iron accumulation and microbleeds in the brain. Acquisition parameters: 12 echoes TE = [2.84, 6.20, 9.56, 12.92, 16.28, 19.64, 23.00, 26.36, 29.72, 33.08, 36.44, 39.80] ms, TR = 44 ms, α = 15°, FOV = 192 x 180 x 144 mm^3^, slice oversampling 11%, 1 mm^3^ isotropic voxels, GRAPPA = 2, 12 min. We will fit a decaying exponential across the echo times to determine the T2^*^ signal at each voxel. This measure is sensitive to vascular pathology that may lead to neurodegeneration. We will use it to detect changes in vascular pathology over the course of the interventions and follow-ups, as well as to assess baseline vascular risk factors that may impact the course of treatment.

### Statistical analyses

Primary analyses will be based on intent-to-treat, not per-protocol. However, we will additionally conduct sensitivity analyses for those participants who completed at least 75% of intervention sessions.

*Analysis 1*. To assess whether MBCT improves symptoms of LLD and quality of life compared to an active control (HEP) over 8 and 26 week samples, we will build generalized mixed models to test the following hypotheses: *1A*. The MBCT group will have a greater reduction in Hamilton Depression Scale (HAM-D17) scores compared to HEP controls, and *1B*. The MBCT group will maintain these improvements at 26-weeks.

*Analysis 2*. Investigate the impact of MBCT on cognitive deficits in LLD we will analyze the Z-scores at initial, 8-week and 26-week follow-up of MBCT and HEP groups using generalized mixed models to test the following: *2A*. Compared to HEP, MBCT will be associated with greater improvements in executive functioning and processing speed scores, and *2B*. The MBCT group will maintain these improvements at 26-weeks.

*Analysis 3:* The neuroimaging analysis is a pilot that will generate effect size estimates for future definitive studies examining neural mechanisms of MBCT in LLD, including the following: *3A*. MBCT patients will be superior to HEP controls with respect to increases in hippocampal volume, increases in default network resting-state functional connectivity, and decreases in executive network resting-state functional connectivity at 8 weeks, and *3B*. The MBCT group will maintain these changes at 26-weeks.

To assess changes in hippocampal volume and functional connectivity, we will implement whole-brain spreading models as in our previous work (Lifshitz et al., 2019), to specifically test for voxels in which the MBCT group exhibits change from baseline to 8 and 26-weeks while the control group does not. To assess functional connectivity, we will implement a standard seed-based analysis using 10 mm seeds positioned in the dorsolateral prefrontal cortex and posterior cingulate cortex, indexing connectivity of the executive network and default network, respectively. Vertex-wise analyses will be performed using the RMINC toolbox (Lerch et al., 2017), as in previous work (Lifshitz et al., 2019). Analyses will consider age, sex, and brain volume/connectivity; clinical/cognitive variables will be considered as independent variables. We will use False Discovery Rate to correct for multiple comparisons (Genovese et al., 2002).

#### Power analysis

We estimate a total sample size of 213 participants randomized, with 170 study completers (20% loss to follow-up) based on effect sizes (ES) estimated from our preliminary data and meta-analyses of adult mindfulness-based interventions RCTs. In our preliminary RCT (Torres-Platas et al., 2019), 8-week MBCT was associated with a 7.9 (SD: 4.4) point improvement on PHQ-9 scores vs. TAU 4.0 (SD: 4.7). Cohen's d = (7.9–4.0)/4.55 = 0.86. Two meta-analyses of mindfulness RCTs (*n* = 35 studies (Hofmann et al., 2010) and *n* = 12 studies (Strauss et al., 2014) found ES (Hedge's g) = 0.50 vs. active control, and ES (g) = 0.41–0.64 vs. TAU in adults with depressive symptoms.

#### Subgroup analyses

We will stratify by sex, gender and site, and reporting outcomes by strata. Sex, along with other baseline characteristics, will also be included as covariates in statistical models to capture their effects on outcomes. We will conduct subgroup analyses on primary outcome (HAM-D depression score) in patients with MCI (MoCA scores 19–25) or normal cognitive functioning (≥26). In addition to site and sex we will describe the following key treatment factors: age, years of education and medical burden. In our *post-hoc* analysis, we will also assess effects, if any, of the following on treatment response: baseline neuroimaging and cognitive biomarkers; anxiety, mindfulness, medication use, ethnicity, socioeconomic status; vascular burden index, age of first depressive episode, and number of lifetime depressive episodes. Because this is a multisite trial, we will fit a site by treatment interaction term to accommodate differential treatment efficacy across sites. We will also stratify smaller and larger groups (8–9 and 10–11) and fit a group size by treatment interaction term to assess differential treatment efficacy among groups of different sizes.

## Discussion

There is an urgent need to test potentially cost-effective, accessible, tolerable, and scalable non-pharmacological treatments for LLD. The proposed study will make use of an active control group, rater-blinding, and validated assessment tools to collect depression and executive function scores and neuroimaging, as well as a number of exploratory outcome measures, which allow us to reliably collect this data with a high degree of fidelity. If MBCT is found to be effective in treating LLD, the collection of neuroimaging data will provide insight on the mechanisms of action of this intervention. MBCT could lower the burden of complications associated with LLD, including disability, increased mortality, suicidality, and risk of cognitive decline (Gildengers et al., 2008; Gilman et al., 2017). In a meta-analysis of younger adult patients, MBCT has been found to be effective in preventing depression relapse (Kuyken et al., 2016), and more recently in treating acute depression and other mental illnesses in real world healthcare settings (Tickell et al., 2020). Since MBCT is delivered in groups and could potentially be adapted into an online intervention, it is scalable in a variety of settings, including primary care. Additionally, future research could examine the cost-effectiveness of MBCT compared to individual psychotherapy or pharmacology for LLD, potentially lowering the direct health-care costs related to LLD (Snow and Abrams, 2016). Elucidating the neural mechanisms of MBCT will pave the road to optimized combination treatment approaches (e.g., with antidepressants or brain stimulation targeting complementary brain circuits). This mechanistic knowledge has the potential to inform patient-tailored precision medicine and target preventative measures for this vulnerable population which has a higher risk for dementia.

## Conclusion

The present protocol will compare the effects of MBCT and an active control group on depressive symptoms, cognitive impairments and neurobiological markers in LLD, and thus inform its use as an adjunct in the treatment of LLD.

## Author contributions

MC, ZS, VN, SR, and MLi: conceptualization and methodology. MB, MLe, HS, and SR: draft preparation and writing and editing. MC, ZS, IR, MSp, AS, DB, MSa, JT, JG, VT, VN, M-AB, SR, MLi, and HS: project administration and management. ED, MC, JG, MLi, and SR: funding acquisition. All authors have substantially contributed to the preparation, critical review, commentary revision, and approval of the manuscript.

## Funding

This project was funded by the Canadian Institutes of Health Research (CIHR; Grant # PJ8-169696 and # PJT-175191), the Fonds de recherche du Québec–Santé (GRQS; Grant # 2022-VIAP-308195), the Jewish General Hospital (JGH) Foundation and charitable donations from the Doggone Foundation. The CIHR, FRQS, JGH and Doggone Foundations had no role in the design of the study or in the writing of this manuscript, collection, and analysis, or interpretation of data.

## Conflict of interest

SR receives a salary award from the Fonds de Recherche de Québec Santé FRQS, is a consultant for AbbVie, and is a shareholder of Aifred Health. HS has a CIHR fellowship award, MITACS fellowship award, and AGE-WELL award. ZS is a cofounder of Mindful Noggin and receives royalties from Guilford Press. The remaining authors declare that the research was conducted in the absence of any commercial or financial relationships that could be construed as a potential conflict of interest.

## Publisher's note

All claims expressed in this article are solely those of the authors and do not necessarily represent those of their affiliated organizations, or those of the publisher, the editors and the reviewers. Any product that may be evaluated in this article, or claim that may be made by its manufacturer, is not guaranteed or endorsed by the publisher.

## Abbreviations

CIRS-G: Cumulative Illness Rating Scale-Geriatric
FFMQ: Five-Factor Mindfulness Questionnaire
fMRI: Functional Magnetic Resonance Imaging
GAD-7: Generalized Anxiety Disorder-7
HAM-D17: Hamilton Depression Rating Scale-17
HEP: Health Enhancement Program
LLD: Late-Life Depression
MBCT: Mindfulness-Based Cognitive Therapy
MCI: Mild Cognitive Impairment
MoCA: Montreal Cognitive Assessment
MRI: Magnetic Resonance Imaging
PHQ-9: Patient Health Questionnaire-9
QoL: Quality of Life
RCT: Randomized Controlled Trial
TAU: Treatment as Usual.
